# Pupillometry as a Measure of Listening Effort in Patients with Bone-Anchored Hearing Systems

**DOI:** 10.3390/jcm11144218

**Published:** 2022-07-20

**Authors:** Wojciech Gawęcki, Katarzyna Krzystanek, Magdalena Węgrzyniak, Renata Gibasiewicz, Małgorzata Wierzbicka

**Affiliations:** 1 Department of Otolaryngology and Laryngological Oncology, Poznan University of Medical Sciences, 60-355 Poznań, Poland; m.wegrzyniak89@gmail.com (M.W.); renatagi@ump.edu.pl (R.G.); otosk2@gmail.com (M.W.); 2 Oticon Medical, 00-133 Warszawa, Poland; kaer@oticon.com

**Keywords:** listening effort, hearing, pupil, pupillometry, bone-anchored hearing systems

## Abstract

The goal of this study is to assess speech comprehension and listening effort by means of pupillometry, in patients with bone-anchored hearing system (BAHS). The study was performed prospectively in a group of 21 hearing-impaired adults, unilaterally implanted with BAHS Ponto. Listening effort was compared in patients wearing two sound processors (Oticon Medical AB, Askim, Sweden): Ponto 3 SuperPower (P3SP) and Ponto Pro (PP). Every patient was invited to two visits, separated by a 3-month break. The first session was to establish the noise level needed to obtain 95% correct sentence recall in the hearing in noise test (HINT), when speech is presented at 70 dB SPL. During the second session, pupillometry, with the use of the above-mentioned conditions, was performed. The mean HINT scores obtained during the second visit were 96.3% for PP and 97.7% for P3SP (*p* = 0.9863). In pupillometry, no significant differences were found for average PPD (peak pupil dilation; *p* = 0.3247), average peak pupil dilation timing (*p* = 0.527) and for pupil dilation growth curves with both processors. The findings of this study suggest that BAHS users allocate similar listening effort with PP and P3SP when processing speech-in-noise at a sound pressure level not saturating Ponto Pro and at a fixed performance level of 95%. This finding applies to the patients who meet the qualification criteria for bone conduction devices and have BC in situ threshold average below 45 dB HL.

## 1. Introduction

The change of pupil size and reactivity in response to certain neurological processes was discovered well over a 100 years ago [1,2]. Large-scale pupillary movements have played a useful role in the daily practice of clinical neurology. If a pupil not reacting to light is detected in a patient with an acute neurological disease, it can be an indication of a traumatic brain injury. A non-reacting pupil is considered to be an important observation and often initiates a number of diagnostic and therapeutic actions [3]. In contrast to large-scale movements, the observation of small-scale, rapid fluctuations in pupillary diameter has aided in understanding the cognitive functions of the human brain. More recently, pupillometry has been applied in psychophysiology to assess cognitive tasks underlined by the changes in the central nervous system [4]. Traditionally, pupil measurements have been performed in a subjective manner by using a penlight or flashlight to evaluate pupil reactivity manually [3]. Nowadays, while hand-held clinical pupillometers are used for neurology and emergency medicine, more advanced automated video-based eye trackers are popular tools in research. They allow for accuracy in measuring pupil size, as well as provide adjustable recording time and connectivity with popular experimental software [5]. In recent years, the interest in using pupillometry to assess listening effort has increased substantially within the fields of audiology and cognitive hearing science [6,7,8].

Listening effort is one of the most common problems people with hearing loss must face, especially in a noisy environment. In challenging listening situations, hearing-impaired people are more fatigued after an hour of conversation than normal-hearing people. This is because the cognitive processes required during speech comprehension in these individuals are associated with more effortful listening [9]. Listening is always associated with the use of cognitive processing, especially in acoustically demanding environments. When speech understanding is degraded by background noise or hearing loss, the listener becomes more reliant on working memory processes to fill in the missing information [10]. The listener is trying to associate the unclear words that he has heard with the words that he already knows (i.e., the words that are stored in his memory) and build a logical sentence using his cognitive abilities [11]. However, when the listening task becomes more demanding, as for most hearing-impaired people, the associated cognitive effort may become overwhelming and lead to tiredness and frustration [12].

The primary means to assess the listening abilities of an individual with hearing loss is a speech intelligibility performance test, the first of which was developed over 70 years ago [13]. Nevertheless, just measuring speech intelligibility is not sufficient to evaluate listening effort in background noise. This is because a listener can compensate for the increased difficulty in a task by increasing the amount of effort, and thus the change in the performance may not become apparent. Combining behavioural measures, such as speech intelligibility testing, with a method evaluating the cognitive load, such as pupillometry, can provide valuable information about the actual listening effort that a person experiences [5,14].

The attempt to decrease listening effort in hearing-impaired people has been the major focus for hearing aid manufacturers in the last decade. They have been constantly trying to improve their devices by advanced signal processing, in order to reduce the background noise and enhance the target speech signal that reaches the listener. The concept of assessing listening effort and cognitive load in hearing-impaired patients has been studied quite extensively in recent years [6,7,8,15,16,17,18,19]. While several studies have investigated listening effort in patients with sensorineural hearing loss [6,7,8,15,16,17,18,19], only one study [6], to our knowledge, evaluated listening effort in patients with a conductive or mixed hearing loss wearing a bone-anchored hearing system (BAHS). A BAHS is a device that transmits sound energy to the inner ear through vibration of the skull, bypassing the outer and the middle ear, which are impaired and thus unable to conduct the sound.

The goal of this study is to assess speech comprehension and listening effort by means of pupillometry, in patients with BAHS. More specifically, the aim is to compare listening effort for patients wearing two sound processors (Oticon Medical AB, Askim, Sweden): Ponto 3 SuperPower (P3SP) and Ponto Pro (PP), at an ecologically valid sound pressure level of 70 dB SPL and at a fixed performance level of 95%. The experimental conditions were designed as an extension of the work of Bianchi et al. [6], where listening effort was also evaluated during a speech-in-noise test for BAHS patients wearing three different sound processors, including PP and P3SP. The researchers aimed at verifying whether a difference in maximum force output (MFO) level and MFO algorithm in the three sound processors would be reflected in differences in listening effort, estimated via pupillary response [6]. The MFO determines the maximum output level that can be transmitted by the sound processor without distorting the signal. The higher MFO in a super-power BAHS will saturate at higher input levels, giving rise to fewer artifacts in the signal at medium to high speech levels depending on the amount of amplification in the device. The researchers concluded, in accordance with their hypothesis, that the participants allocated significantly lower listening effort with the P3SP than with the PP as a consequence of a higher MFO and a multichannel MFO algorithm in the P3SP, resulting in fewer saturation artifacts in the signal [6]. This result was obtained when fixing the speech level for each participant to a rather high sound pressure level such that the PP sound processor would be in saturation in the entire frequency range (but not the P3SP). In a second experiment (“condition 2”), the researchers decreased the speech level, as well as the noise level, by 5 dB to evaluate listening effort for a speech signal with fewer saturation artifacts. Despite still obtaining a difference in overall pupil dilation between P3SP and PP, the researchers were not able to unequivocally determine whether the difference in listening effort obtained in this second condition was due to more saturation artifacts in PP versus P3SP or more audibility limitations in PP versus P3SP.

In this study, we are extending the work of Bianchi et al. by introducing a condition with a fixed speech level of 70 dB SPL—a value similar to the average level in condition 2 from Bianchi et al. (71 dB SPL)—while controlling for performance to be at 95% with PP. Hence, we would rule out the audibility limitation and verify whether we still observe a difference in pupil dilation at a speech level of 70 dB SPL. Another reason for choosing this level is that it reflects sound pressure levels that hearing-impaired people are much more likely to encounter during conversations in real-world noisy environments [20,21,22]. We anticipated that if a larger pupil dilation was observed with PP than with P3SP while controlling for performance (95% correct speech intelligibility), then the difference in listening effort between the two sound processors would be ascribed to more saturation artifacts in PP versus P3SP, even at an ecologically valid speech level of 70 dB SPL.

## 2. Materials and Methods

### 2.1. Study Design

The study was performed prospectively in a group of 21 hearing-impaired adults, unilaterally implanted with BAHS Ponto. Every patient was invited to two visits, separated by a 3-month break. The first session was to establish the noise level needed to obtain 95% correct sentence recall when speech is presented at 70 dB SPL for measurement of pupil dilations. During the second session pupillometry, with the use of the above-mentioned conditions, was performed. The investigation was approved by the local Ethics Committee (decision number 470/19).

### 2.2. Participants

The participants were recruited from the BAHS database of our department. They have bilateral conductive or mixed hearing loss and were experienced BAHS users with at least 5 years of wearing the Ponto device. All of them were unilaterally implanted and there were no adverse events observed since the operation. Ponto users with single-sided deafness were not included in the study. There were 21 native Polish participants—9 males and 12 females—between 23 and 77 years old (mean 58 years; Table 1). None of the patients had eye or brain disease or surgery that might have influenced the results of the study.

### 2.3. Devices

In this study, two types of BAHS sound processors were used: Ponto Pro (PP released in 2010, which is an updated version of PP from 2009, Oticon Medical AB, Askim, Sweden) and Ponto 3 SuperPower (P3SP, available since 2016; Oticon Medical AB, Askim, Sweden). Both PP and P3SP devices have the same maximum force output algorithm (multichannel), but differ in maximum force output (MFO) level, which is lower in PP (which will result in a low dynamic range especially at low frequencies) and higher in P3SP. In order to ensure that the difference between the sound processors was restricted to the difference in MFO level as much as possible, no fine-tuning was performed during fitting. To this end, noise reduction was turned off, directionality was set to omnidirectional mode, and the volume and mute settings were turned off. P3SP and PP are designed for listeners with hearing loss up to and including 65 and 45 dB HL, respectively. Four Ponto processors were used for measurements for all participants—two PP devices: left (SN: 47980772) and right (SN: 49798749) and two P3SP devices: left (SN: 55217460) and right (SN: 55382885).

### 2.4. Experimental Approach

Every patient was invited to two visits, separated by a 3-month break, that lasted an hour each.

At the first session, two measurements were preformed: feedback measurement and BC in situ threshold, both with the P3SP sound processor. The gain settings were prescribed according to the modified NAL-NL1 prescription used in Ponto. The gain settings from the P3SP were then transferred to PP, to ensure that the settings were similar in both processors. After the fitting, the subjects performed a speech intelligibility test with Polish Hearing In Noise Test (HINT) sentences [23] with PP sound processor to measure the individual SNR corresponding to 50% (SRT50) and 80% (SRT80) correct performance with the PP. Two lists with 20 sentences in each were presented to the participants. The first list was the test list to measure the SRT50, and the second list was the test list to measure the SRT80. During these measurements, the speech signal was set at 70 dB SPL. To adjust the noise level in the case of the SRT80 measurement, a decrease of 3.2 dB was applied for if the sentence was incorrectly repeated (0 words correct) and an increase of 0.8 dB if the sentence was repeated correctly (for all words correct). For SRT50, the steps were 2 and 2 dB, respectively. The interval size was doubled for the first four sentences. The noise level was estimated individually for each listener to lead to 95% (SRT95) correct performance with PP, which was used as baseline (results listed in Table 1). To this end, a psychometric function was fit to the SRT50 and SRT80 to calculate the SRT95 [6]. In order to ensure that 95% correct words was reached even when the processor was in saturation the PP was used as a reference point.

The second visit consisted of pupillometry measurements conducted during a speech intelligibility test with HINT sentences. The sentences were presented at the fixed speech level of 70 dB SPL and noise presented at the estimated level that provides SRT95 for each participant. Pupil dilation was measured while each participant was listening to one out of three lists (20 sentences per processor) in a randomized manner, wearing one randomly chosen processor. Afterwards, the other processor was tested and another random list was presented to the participant. The participants were asked to listen to and repeat each sentence after the background noise has stopped. There was a break introduced for each participant while switching the processors.

During both visits, the opposite ear of all participants was muffled by means of soundproof headphones to ensure that no sounds were perceived through this ear and only contribution from the implanted side was tested. For most of the patients, the study conditions were comfortable, but a few of the patients reported a slight inconvenience wearing earmuffs, eye tracking goggles and a BAHS processor at the same time. Listeners arrived at the clinic from different locations and using various means of transportation on the pupillometry measurement day. The patients were invited to two separate visits, because each visit was quite an extensive measurement day for them. We needed to measure their SRT50 and SRT80 prior to estimating SRT95 and we did not want these measurements to influence their performance during the final pupillometry test, as it might have caused more fatigue. There were no alterations in the auditory thresholds within this period, which we have confirmed by making BC in situ measurements again after the 3-month period.

### 2.5. Test Set Up

**Figure 1:**
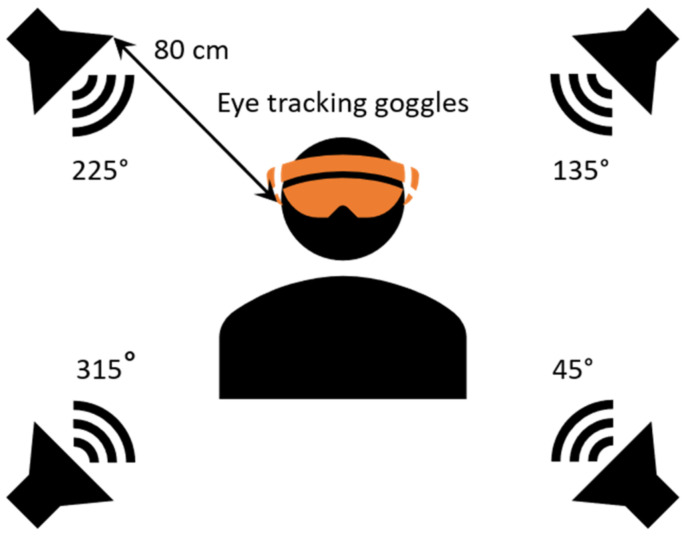
The loudspeaker arrangement used in this study. They were placed in four corners of the booth: the front ones were presenting target speech and the noise was played from the loudspeakers in the back of the listener.

The experiment performed in this study was carried out in a soundproof booth. Four loudspeakers (Ecler, Neec Audio, Barcelona, Spain) were placed in each corner of the booth and in a 80 cm distance from the listener at 45°, 135°, and 225° and 315° azimuth (Figure 1). The target speech signal was presented from the loudspeaker at the 45° and 315° azimuth position, while the noise was presented from the loudspeakers at 135° and 225° azimuth. The audiometer used in the study was the AC40 model from Interacoustics (Middelfart, Denmark). The listeners were sitting in the centre with eye-tracking Goggles on (Pupil Labs GmbH, Berlin, Germany) and looking in front of them. For each processor, a list of 20 Polish HINT sentences [23] was presented in a speech-shaped noise.

### 2.6. Pupil Data Analysis

Pupil data analysis was performed similarly to previously described procedures [6,8]. At the beginning of the measurement the listeners were given some time to accustom to the test. The first five sentences were discarded.in order to remove any bias related to this accommodation time. Pupil curves obtained for sentences between the 6th and 20th were then averaged to obtain the mean dilation for each participant. For each listener, the mean pupil dilation was calculated by averaging the processed pupil traces, starting from 1 s before sentence onset to 4.66 s after sentence onset as it was the average noise offset. The raw data were first resampled to 60 Hz to ensure uniform sampling rate. Then, blinks were detected using the median absolute deviation [24]. Datapoints ranging from 35 ms before the blink to 50 ms after the blink were also discarded. The remaining data 2.5 × standard deviation away from the mean was also removed [5], as well as groups of datapoints of a length inferior to 40 ms. Data with more than 35% of the datapoints removed were discarded. Interpolation was performed using cubic spline if there were enough available data on both sides of the detected blink; linear interpolation was used otherwise. Finally, the data points were filtered using a moving average window of 50 ms. All subjects had less than 50% discarded trial data. A baseline value was computed for each sentence as the mean pupil diameter measured during the one-second time range before the sentence onset-during the noise presentation. This value was further subtracted from each pupil dilation curve to obtain a baseline-corrected pupil curve.

### 2.7. Statistical Analysis

#### 2.7.1. HINT Test Scores

In order to compare HINT test scores of all participants using PP or P3SP, a Wilcoxon rank sum test with continuity correction was performed, using a statistical package in R-studio: wilcox.test {stats}.

#### 2.7.2. Peak Pupil Dilation

The peak pupil dilation (PPD) was extracted from the averaged curve for each participant and each condition, as well as its timing. The PPD was defined as the maximum pupil dilation within the time interval between the sentence onset and the average noise offset. In order to compare the PPD values of all participants using PP or P3SP, a Wilcoxon rank sum test with continuity correction was performed, using a statistical package in R-studio: wilcox.test {stats}.

#### 2.7.3. Growth Curve Analysis

To model the pupil dilation as a function of time, growth curve analysis (GCA) was applied [6,8,25] using a mixed-linear model with subject as a random factor. The data from the averaged curves’ first inflation point, to the end of the considered time range was modelled using a polynomial orthogonal basis up to the 3rd order. The modelling was done using MATLAB (The Mathworks, 2016) with a Satterthwaite correction on the estimated levels. The resulting model’s equation was:

$$\begin{array}{cc} pupil\;dilation & =Proc\times \left(1+poly1+poly2+poly3\right)\\ & +(1+poly1+poly2+poly3\;|\;TP) \end{array}$$

where *Proc* represents the conditions Ponto Pro or Ponto 3 SuperPower and *TP*–the study’s participants. The hypothesis was tested at a significance level of 5% (*p* < 0.05).

## 3. Results

The populational as well as audiological data for all 21 participants in the study are presented in Table 1.

**Figure 2:**
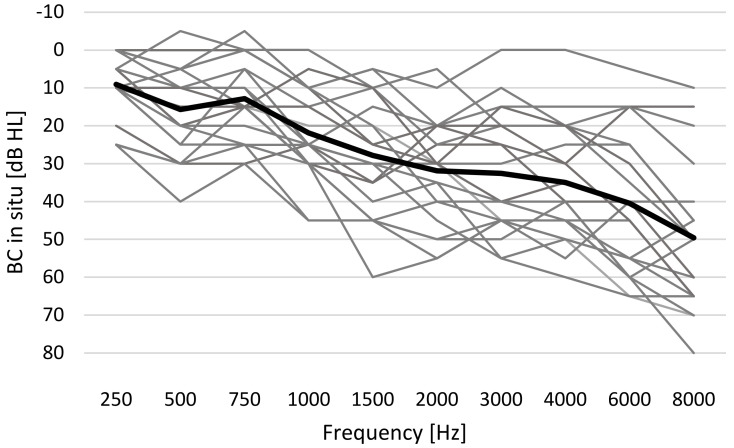
Bone conduction (BC) hearing thresholds measured in situ for the 21 participants (mean BC threshold is shown with the thick line). Ponto 3 SuperPower (P3SP) was used to acquire the data.

The individual bone conduction (BC) hearing thresholds, which were measured in situ through the sound processor, are presented in Figure 2. The bone conduction pure tone average (BC-PTA, Table 1) was calculated for the BC in situ thresholds at 0.5, 1, 2, and 3 kHz for all the participants and was within the fitting range of both sound processors, i.e., ≤45 dB HL.

The measurements of 18 patients were analyzed. Three patients were excluded from further analysis (see Table 1) due to the fact that the individually adjusted noise levels to obtain SRT95 were below or very close to their bone conduction pure tone average (BC-PTA). Thus, those individuals could in practice not hear the noise, and thereby were not conforming to the criteria of the study.

### 3.1. HINT

The mean HINT scores obtained during the second visit (with the sentences presented at the fixed SRT95 for each participant) were compared between PP and P3SP and were equal to 96.3% and 97.7%, respectively. They resulted in a statistically non-significant difference (*p*-value = 0.9863) in performance between the two Ponto devices. These results were in accordance with our initial design, where the listeners had their performance levels calibrated to 95%.

### 3.2. Pupillometry

**Figure 3:**
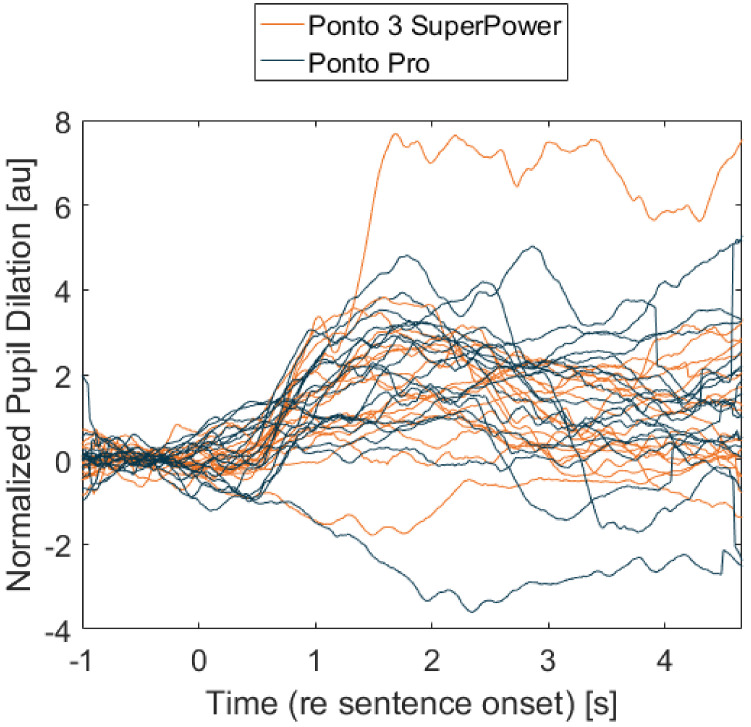
Mean pupil response for each participant (*n* = 18). The orange curve corresponds to the Ponto 3 SuperPower and the blue curve to the Ponto Pro.

**Figure 4:**
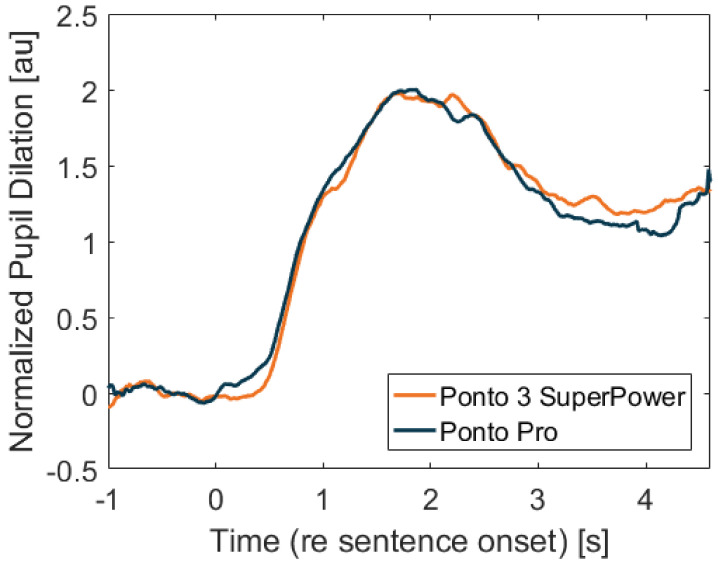
Mean pupil responses averaged across participants (*n* = 18). The orange curve corresponds to the Ponto 3 SuperPower and the blue curve to the Ponto Pro.

**Figure 5:**
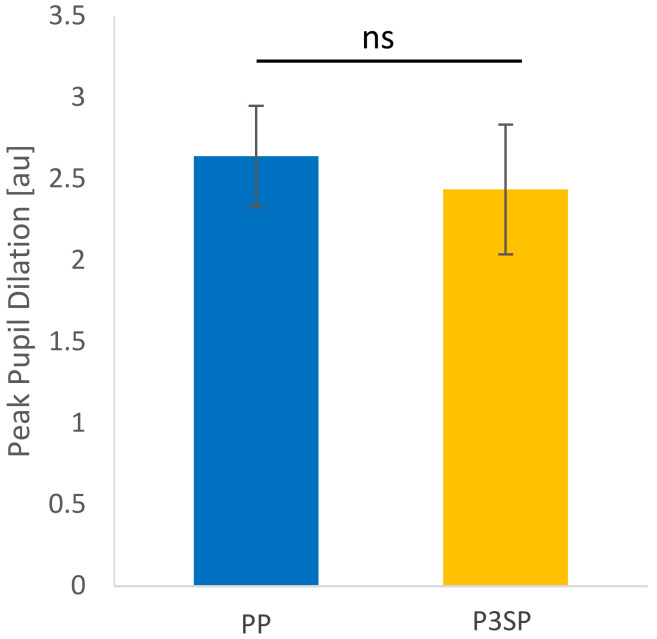
Average peak pupil dilation of all test participants wearing Ponto Pro and Ponto 3 SuperPower. There is no significant (ns) difference between the two processors (*p*-value = 0.2892). Error bars represent standard error.

**Figure 6:**
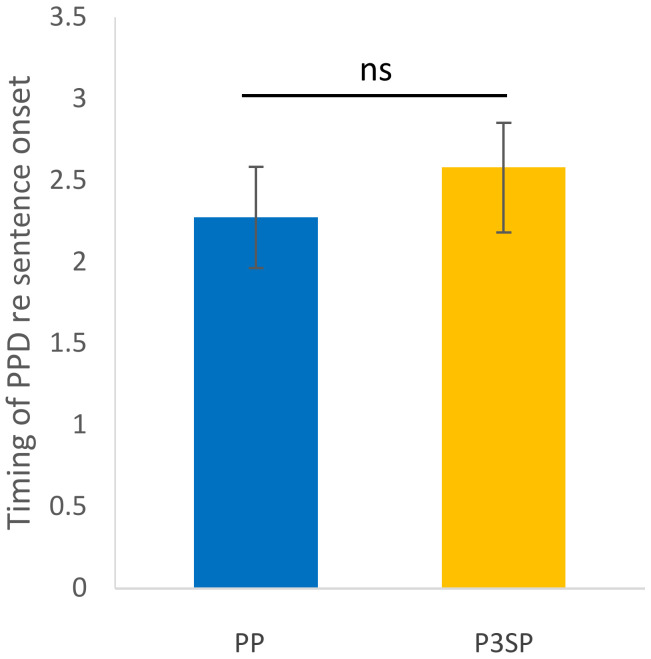
Average peak pupil dilation timing with respect to the sentence onset of all test participants wearing Ponto Pro and Ponto 3 SuperPower. There is no significant (ns) difference between the two processors (*p*-value = 0.527). Error bars represent standard error.

Each of the participant’s average pupil dilation curves are presented in Figure 3, and the resulting average per condition is shown in Figure 4. It can be observed that the two curves are very similar, following the same pattern. The participants’ average PPD is presented in Figure 5 and there is no significant difference between the two processors (*p*-value = 0.3247). The average peak pupil dilation timing is depicted in Figure 6 and there is also no significant difference between the two processors (*p*-value = 0.527).

Table 2 presents the results of the growth curve analysis performed as previously described [6,8,25]. Four orthogonal terms were estimated for each processor to analyze the pupil dilation data, and they correspond to the polynomial order of: 0 = intercept, 1 = slope (linear), 2 = quadratic and 3 = cubic. According to Mirman et al., the intercept term indicates the average height of the curve (equivalent to area under the curve), the slope term indexes the overall angle of the curve, the quadratic term reflects the rate of inclining and declining around a central inflection point, and the cubic term corresponds to the steepness of the curve around inflection points. Although the linear term is significantly different from one processor to the other, its value for either condition is not significantly different from 0, and thus it does not contribute to the model. Thus, only the intercept, quadratic and cubic terms were used to estimate differences in pupil dilation curves with both processors. The resulting *p*-values of the three terms indicate that there is no significant difference between the two devices.

## 4. Discussion

The aim of this study was to evaluate the listening effort of hearing-impaired patients implanted with BAHS and to compare the effort allocated to process speech in noise while using two different sound processors: Ponto Pro and Ponto 3 SuperPower. We have designed this work as a continuation of a study by Bianchi et al. [6], in which these two processors, among others, were the subject of comparison. In the study by Bianchi et al. the speech was fixed at an individual level where PP was in saturation, but P3SP would not reach saturation in its second MFO band, and noise level was estimated so that SNR would lead to 95% correct performance with the PP (“condition 1”). However, in the second condition, where both signal and noise levels were lowered by 5 dB, a decline in performance for participants with higher BC-PTA (bone conduction pure tone average) appeared among the listeners, suggesting an audibility limitation. That study design did not allow one to determine whether the difference in overall pupil dilation obtained in condition 2 was the result of fewer artifacts in P3SP versus PP or more audibility limitations in PP versus P3SP.

In the present study, we have extended the work of Bianchi et al. [6] by fixing the speech level at 70 dB SPL while controlling for performance to lead to 95% correct HINT scores with PP. In this way, we would ensure that the signal was audible to the listeners throughout the experiment. We have observed that the pupil responses were similar between the two sound processors, both in terms of PPD and morphology of the pupil traces, indicating that the participants allocated similar listening effort when processing speech-in-noise with the two sound processors. This finding suggests that there are no large differences in terms of saturation artifacts between the two sound processors at a speech level of 70 dB SPL, at least for the patients included in this study (with an average BC hearing loss equal to or below 45 dB HL, hence for patients within the fitting range of both sound processors). These considerations cannot be extended to patients with larger hearing losses, for which a higher amplification is needed, leading to saturation artifacts in PP already at medium speech levels.

The design of this study ensured that the participant could understand 95% of the sentences at a signal level of 70 dB SPL, which allowed us to determine which of the two potential factors was affecting condition 2 in Bianchi et al. (audibility limitations vs. saturation artifacts). The lack of a significant difference between PP and P3SP in this study suggests that audibility limitations were present in condition 2 in Bianchi et al. and were likely driving the difference in overall pupil dilation in the second condition (rather than saturation artifacts in PP).

However, an important aspect to consider when comparing the two studies is that the PP used in this study is different from the PP used in Bianchi et al. [6]. In the current study, an upgraded PP was used with a four-channel MFO algorithm (i.e., it has four frequency bands), in contrast to the single channel MFO algorithm used in Bianchi et al. This means that if one frequency band is in saturation, the remaining bands can still operate with full dynamics. The upgraded PP is more similar in this aspect to the Ponto 3 (P3) from Bianchi et al. [6], which also has a four-channel MFO, but differs in the level of MFO. In Bianchi et al., it was demonstrated that the overall pupil dilation difference between P3 and P3SP was about 18% in condition 1 at high speech levels [6]. If saturation artifacts would have been present also at 70 dB SPL, a comparable difference could have been observed in our study, given the similar technical parameters between the upgraded PP and P3. However, the lack of a statistically significant difference in pupil dilation between PP and P3SP in our study can be explained by the fact that the PP (with the multichannel MFO algorithm) did not reach saturation at 70 dB SPL (at least not throughout the whole frequency range). This suggests that the amount of artifacts in the output speech signal was likely similar between the two sound processors, or not sufficiently different to be captured by pupil responses.

This study was performed in the test conditions where all advanced signal processing functions were switched off. Therefore, we cannot exclude a difference in listening effort between PP and P3SP in real-life circumstances. In everyday life, the listeners would have several automatic features turned on in their processors, such as directionality or noise reduction, that might have an effect on audibility and speech understanding. In the future, it might be interesting to carry out additional experiments in order to improve our understanding of the advantages and limitations of the different sound processor features. For instance, one might investigate how and to what degree the difference in PP and P3SP’s advanced signal processing contributes to the listening effort.

### Limitations

A possible limitation of the present study, which might have affected pupil dilation, is the diversity of the participants. For example, the age span of the listeners was substantial, i.e., 23–77 years old, and it has been demonstrated before that pupil size decreases with age [26]. The intellectual and cognitive abilities of the patients might have been also quite different, as can be concluded from the initial interviews (participants come from different backgrounds, etc.). The considerable differences in pupil responses visualized in Figure 3 are also indicative of a cognitive variety among the participants. The listeners BC-PTA is very different—it stretches from 5 to 38.8 dB HL and, similarly, the adjusted SNR ranges from 1.6 to 17.2 dB (Table 1). Additionally, the listeners arrived to the clinic from different locations and using various means of transportation on the pupillometry measurement day, which might have had an impact on their overall fatigue and thus their performance on the test day. Moreover, a few of them reported a slight inconvenience wearing earmuffs, eye tracking goggles and a BAHS processor at the same time, which might have influenced their performance as well. Thus, it would be beneficial to recruit a more homogenous cohort of patients and optimize the conditions to be more similar for all participants.

Another aspect to be considered is that the noise needed to be decreased quite substantially to ensure that every participant reached 95% of speech comprehension. Three subjects (the ones not included in the study) would never have been able to obtain SRT95 when noise was present (audible for them, Table 1). The remaining subjects (*n* = 18) required to have on average an SNR of 11 dB (calculated from data in Table 1) to understand 95% of the sentences. This finding suggests that there would be many listening situations with background noise, where their speech comprehension was less than 95%. In other words, our cohort of patients would require a rather low-noise conversation environment to be able to understand the speech completely. The difference between the experimental set-up applied in Bianchi et al. and our work was the type of noise used in the HINT tests. It was a babble noise and a speech-shaped noise, respectively. Despite this modification, we do not expect that it might have an impact on our results, since PP can saturate with both types of noise.

The pupillometry measurements conducted in the presented study have led us to an observation that certain eye anatomy features (the outer eye parts) may be challenging for the eye-tracking goggles. More specifically, we have noted that in the case of patients with drooping eyelid the camera had difficulties with setting the focus on a pupil, since the eyelid was covering it to a certain extent. There was also a case including a participant with a mole protruding up from the lower eyelid, which also created a challenge in setting the camera focus right. Hence, it might be a benefit in the future experiments to use these features as criteria excluding the listeners from participating in a similar study, so that the measurements are less biased.

## 5. Conclusions

The findings of this study suggest that BAHS users allocate similar listening effort with Ponto Pro and Ponto 3 SuperPower when processing speech-in-noise at a sound pressure level not saturating Ponto Pro and at a fixed performance level of 95%. This finding applies to the patients who meet the qualification criteria for bone conduction devices and have a BC in situ threshold average below 45 dB HL (for patients within the fitting range of both sound processors).

## Figures and Tables

**Table 1:** Populational information and audiological data of the 21 participants. BC-PTA (bone conduction pure tone average) was calculated as a mean BC in situ thresholds at 0.5, 1, 2, and 3 kHz. The SNR (signal-to-noise ratio) was calculated for fixed speech level of 70 dB SPL and individually adjusted noise levels to obtain SRT95 with PP. Participants with noise level marked with a star (*) were excluded from further analysis due to the fact that these levels were below or very close to their bone conduction pure tone average (BC-PTA).

#	Gender	Ear Implanted	Age (Years)	BC-PTA (dB HL)	Noise Level (dB SPL)	SNR (dB)	HINT Scores	
							PP	P3SP
1	M	L	51	23.8	63.7	6.3	100	98.4
2	M	R	67	28.8	53.7	16.3	96.3	98.5
3	M	R	72	33.8	41.1 *	28.9	100	100
4	F	L	58	25.0	55.6	14.4	100	96.9
5	F	L	62	40.0	23.8 *	46.2	88.9	85.5
6	M	L	40	12.5	53.3	16.7	100	100
7	F	R	47	25.0	61.3	8.7	100	92.6
8	M	L	51	20.0	63.6	6.5	90	100
9	M	L	67	33.8	52.8	17.2	96.3	98.4
10	F	R	77	33.8	57.9	12.1	100	100
11	F	L	63	41.3	59.9	10.1	72.2	96.8
12	F	L	49	45.0	40.9 *	29.1	98.5	96.3
13	M	L	62	21.3	54.3	15.7	100	100
14	F	L	70	28.8	59.9	10.1	100	93.8
15	F	L	23	6.3	57.6	12.4	100	100
16	F	L	67	16.3	57.7	12.3	100	100
17	M	L	73	25.0	58.6	11.4	98.1	100
18	F	R	72	38.8	59.0	11	88.9	98.5
19	F	L	48	5.0	68.1	1.9	100	100
20	M	R	37	15.0	65.1	4.9	100	98.1
21	F	R	63	17.5	68.4	1.6	92.6	98.4

**Table 2:** Growth curve analysis output summary of the intercept, linear, quadratic, and cubic terms for each of the processors, obtained by changing the reference in the model. The corresponding *p*-values < 0.05 for Ponto 3 SuperPower or Ponto Pro indicate a significant difference from 0; significant difference between the processors is indicated by a *p*-value < 0.05.

	Ponto 3 SuperPower		Ponto Pro		Difference
Model Estimates	Estimate	*p*-Value	Estimate	*p*-Value	*p*-Value
Intercept	1.361	<0.001	1.340	<0.001	0.264
Linear	1.402	0.405	0.341	0.838	<0.001
Quadratic	−5.443	<0.001	−4.922	<0.001	0.091
Cubic	4.950	<0.001	5.247	<0.001	0.336

## Data Availability

The data presented in this study are available on request from the corresponding author.
